# Auditory-Motor Rhythms and Speech Processing in French and German Listeners

**DOI:** 10.3389/fpsyg.2017.00395

**Published:** 2017-04-11

**Authors:** Simone Falk, Chloé Volpi-Moncorger, Simone Dalla Bella

**Affiliations:** ^1^Institut für Deutsche Philologie, Ludwig-Maximilians-UniversityMunich, Germany; ^2^Laboratoire Parole et Langage, UMR 7309, Centre National de la Recherche Scientifique, Aix-Marseille UniversityAix-en-Provence, France; ^3^Laboratoire Phonétique et Phonologie, UMR 7018, CNRS, Université Sorbonne Nouvelle Paris-3Paris, France; ^4^EuroMov, University of MontpellierMontpellier, France; ^5^Institut Universitaire de FranceParis, France; ^6^International Laboratory for Brain, Music, and Sound ResearchMontreal, QC, Canada; ^7^Department of Cognitive Psychology, Wyższa Szkoła Finansów i Zarządzania w Warszawie (WSFiZ)Warsaw, Poland

**Keywords:** speech perception, multisensory rhythm, verbal processing, temporal expectancies, temporal prediction

## Abstract

Moving to a speech rhythm can enhance verbal processing in the listener by increasing temporal expectancies (Falk and Dalla Bella, 2016). Here we tested whether this hypothesis holds for prosodically diverse languages such as German (a lexical stress-language) and French (a non-stress language). Moreover, we examined the relation between motor performance and the benefits for verbal processing as a function of language. Sixty-four participants, 32 German and 32 French native speakers detected subtle word changes in accented positions in metrically structured sentences to which they previously tapped with their index finger. Before each sentence, they were cued by a metronome to tap either congruently (i.e., to accented syllables) or incongruently (i.e., to non-accented parts) to the following speech stimulus. Both French and German speakers detected words better when cued to tap congruently compared to incongruent tapping. Detection performance was predicted by participants' motor performance in the non-verbal cueing phase. Moreover, tapping rate while participants tapped to speech predicted detection differently for the two language groups, in particular in the incongruent tapping condition. We discuss our findings in light of the rhythmic differences of both languages and with respect to recent theories of expectancy-driven and multisensory speech processing.

## Introduction

In everyday communication and interaction, we often experience our sound environment through movement. We sway with music, move our eyebrows, head, or body while participating in a conversation, we gesture while speaking (e.g., McNeill, 1992; Janata et al., 2012). In particular, rhythmic sounds such as music featuring a regular beat or speech with a metrical structure (e.g., as in rhymes or poetry) are often accompanied by coordinated rhythmic movement (e.g., Ong, 2002; Maes et al., 2014). Parents stimulate their infants via concurrent rhythmic movements in verbal games and nursery rhymes (e.g., Stern, 1974; Opie and Opie, 1997). Older children utter words and move simultaneously during rhyme games and song by clapping their hands or stamping their feet (e.g., Opie and Opie, 1988). Past research has shown that rhythm facilitates multisensory coordination and temporal perception (Manning and Schutz, 2013, 2016). In return, aligning motor to verbal rhythms facilitates verbal processing (Falk and Dalla Bella, 2016). The mechanisms underlying the beneficial outcomes of aligned auditory-motor rhythms, particularly in the verbal domain, are still unclear. Here we aim at contributing to this issue by examining auditory-motor alignment and its effects on verbal processing in two rhythmically diverse languages, namely French and German.

There is evidence that cognitive benefits of auditory-motor rhythms can be driven by temporal expectancies. In general, when we expect something to happen at a certain time, we attend more to that particular moment than to another time (e.g., Large and Jones, 1999). Auditory rhythms such as metrical speech or music with a salient beat structure feature accent patterns of syllables and notes that recur (quasi-) periodically in time (e.g., Lehiste, 1977; London, 2004) and are perceived as highly regular. An “accent” refers to a prominent syllable or note marked by the expansion of pitch, intensity, duration or other heightened acoustic properties (e.g., articulatory clarity in speech). Thanks to the recurrence of these accent patterns, a predictable temporal structure (rhythm) emerges across several timescales. That these auditory rhythms enhance attending is supported by both behavioral and psychophysiological evidence (Jones et al., 2002; Ellis and Jones, 2010). EEG studies show that periodic sequences of tones and syllables evoke larger amplitudes and shorter latencies of attention-related brain potentials (i.e., the P3b) than sequences with aperiodic rhythmic organization (Schwartze et al., 2011; Otterbein et al., 2012). In addition, higher attending to temporally predictable sounds is underpinned by neural oscillatory activity that phase-locks to rhythmic periodicities in the auditory signal at hierarchically nested frequencies (e.g., Lakatos et al., 2008; Fujioka et al., 2010; Peelle et al., 2013). Independently of the exact neural basis of rhythmic attending, which is still debated, several studies point to behavioral advantages of temporally predictable sound sequences in both speech and music. In metrical speech, information is better processed and remembered (Quené and Port, 2005; Dilley and McAuley, 2008; Roncaglia-Denissen et al., 2013), in particular when it occurs on accented compared to unaccented syllables (Pitt and Samuel, 1990; Zheng and Pierrehumbert, 2010; Falk and Dalla Bella, 2016). In sum, these findings lend support to the conclusion that temporal expectancies driven by an auditory rhythm in speech may help enhancing the perceptual salience of prominent syllables, and, thereby, facilitate verbal processing at these points in time.

Movement coordinated with the rhythm of sound sequences can also modify the way listeners attend to and encode auditory stimuli. This has been shown with musical material (e.g., Phillips-Silver and Trainor, 2005). For example, synchronous motor activity with periodic tone sequences enhances the amplitude of attention-related brain potentials (Schmidt-Kassow et al., 2013; Conradi et al., 2016). In another EEG study, Chemin et al. (2014) showed that participants moving their hand to either a binary or ternary beat pattern of rhythmically ambiguous music encoded the rhythm of the sound through the rhythmic movement pattern. When the same participants listened to the music later, their brain responses were enhanced at exactly those points in time when their movements had previously occurred. This finding is compatible with the “active sensing” framework which posits that the motor system shapes processing of sensory information by linking auditory information to temporal predictions generated by action planning and execution (see Morillon et al., 2015, for an overview). The results of Chemin et al. (2014) also fit with the common coding theory (Prinz, 1990; Hommel et al., 2001), which states that predictions associated with motor action planning and execution are jointly coded with auditory predictions in the cognitive system (see also Maes et al., 2014, for a review).

While these theoretical approaches have been used to explain the role of rhythm and auditory-motor coupling in music, evidence for multisensory rhythmic effects in speech is still scarce. In a recent study, we investigated the effects of shared predictions generated by a temporally aligned auditory-motor rhythm on speech perception (Falk and Dalla Bella, 2016). Participants were cued to align or misalign a motor rhythm (i.e., finger tapping) with the accented syllables of a metrically structured German sentence. The sentence was then repeated without tapping and the participants detected a verb change occurring either on accented or unaccented syllables. Results showed that participants were most successful in detecting the changes when their finger taps were aligned with the accented syllables compared to misalignment, and when the changes also occurred on accented syllables. Thus, finger tapping aligned to accented syllables is a critical factor leading to improved verbal performance. These findings suggest that the benefits of aligning motor and auditory rhythms may be driven by overlapping temporal expectancies. The result would be a maximal enhancement of attentional resources at the rhythmic reference points (i.e., accented syllables) in the speech signal.

One of the open questions is whether the observed auditory-motor effects are confined to German prosody, or generalize to other languages. German is described as a “stress”-language (Jessen, 1999; Wiese, 2000). At the lexical level, each word is learned with a specific pattern of stressed and unstressed syllables. Thus, stress accents may distinguish meaning and thereby fulfill a *contrastive* function in German. In the context of spoken discourse, the German stress-accents also have a *cumulative (i.e., head-marking)* function within their prosodic domain (i.e., feet, prosodic words, and phrases, intonation phrases, etc.), resulting in complex metrical relations between subsequent accents that mark different hierarchical levels, similar to English (Liberman and Prince, 1977; Hayes, 1995). In the German stress system, de-stressing as well as heightened stress are flexibly used to signal communicative deviations from syntactic and semantic default interpretations (e.g., to signal focus; Féry and Ishihara, 2008; Féry and Kügler, 2008). Analysis of the profile of stress-accents (i.e., their position and strength) as a key to comprehend the meaning of words and nested discourse units is common in German listeners. Hence, the German prosodic system may be particularly well-suited to drive rhythmic expectancies and to lead to the ensuing benefits on verbal processing. Here we tested whether predictions driven by auditory-motor rhythms extend to French, a language that differs substantially from the prosody of German. French is most often described as lacking lexical stress (Rossi, 1980). Indeed, accentuation in French has predominantly a *demarcative (i.e., boundary-marking)* function within the prosodic domain of the accentual phrase (AP, Vaissière, 1991; Jun and Fougeron, 2000, 2002). Primary accents in French are assigned to the last syllable (except Schwa-syllables) of a phrasal segment, thereby marking the right edge of the AP. Secondary accents, though not obligatory, can be found toward the left edge of an AP, depending on the number of syllables and on the lexical composition of the phrase (e.g., Astésano, 2001; Welby, 2003, 2006). Hence, accent placement in French is tightly linked to grouping and phrasing, and, ultimately, to syntactic segmentation (e.g., Millotte et al., 2008; Michelas and D'Imperio, 2015). Importantly, compared to German, stress conflicts between words and phrases such as stress clash and shift (e.g., Mengel, 2000; Bohn et al., 2013) are more rare (Post, 2000). Peperkamp and Dupoux (2002) have suggested that because French lacks lexical relevance of accents and has fixed accent placement, French listeners pay less attention to accentual variations as compared to listeners of languages with lexical/variable stress. This intriguing hypothesis is supported by the observation of difficulties in encoding and memorizing stress contrasts (i.e., “stress-deafness”) in native French listeners when learning a foreign language with lexical/variable stress (e.g., Dupoux et al., 2008; Schmidt-Kassow et al., 2011; Domahs et al., 2012; but see also Michelas et al., 2016). Thus, we anticipate that French listeners will attend less to accented syllables while tapping to them than German listeners did. As a result, the benefit of aligning movement and speech rhythms may be less visible in French than in German.

We tested this hypothesis by asking French and German listeners to detect a verbal change in their native language in the context of an aligned or misaligned auditory-motor rhythm. The paradigm is the same as in Falk and Dalla Bella (2016). A second goal was to closely examine whether individual differences in motor performance could predict the success in this task for both languages.

## Materials and methods

### Participants

Thirty-two French-speaking students from the Aix-Marseille University (4 males, *M* = 22.5 years, *SD* = 4.0 years, 3 left-handed) and 32 German-speaking students from the Ludwig-Maximilians-University in Munich (3 males, *M* = 24.6 years, *SD* = 5.5 years, 4 left-handed) took part in the Experiment. The German-speaking group was a subgroup of a larger study (see Falk and Dalla Bella, 2016). None of the participants was a professional musician, although the German speakers overall had more musical experience than the French speakers [French group: 10 participants with music lessons, range = 4–20 years; German group: 27 participants with music lessons, range 2–18 years, Mann–Whitney *U*_(63)_ = 713.5, *p* = 0.001]. Sixteen additional French-speaking students (7 males, *M* = 21.8 years, *SD* = 2.8 years, 4 left-handed and 1 ambidexter, 9 participants with music lessons, range: 1–20 years) and 16 additional German-speaking students (9 males, *M* = 25.0 years, *SD* = 3.3 years, all right-handed, 11 participants with music lessons; range: 1.5–12 years) participated in a perceptual control experiment (“Baseline”). All the participants gave informed written consent. The study was approved by the Ethics Committee of Aix-Marseille University.

#### Materials

**Figure 1:**
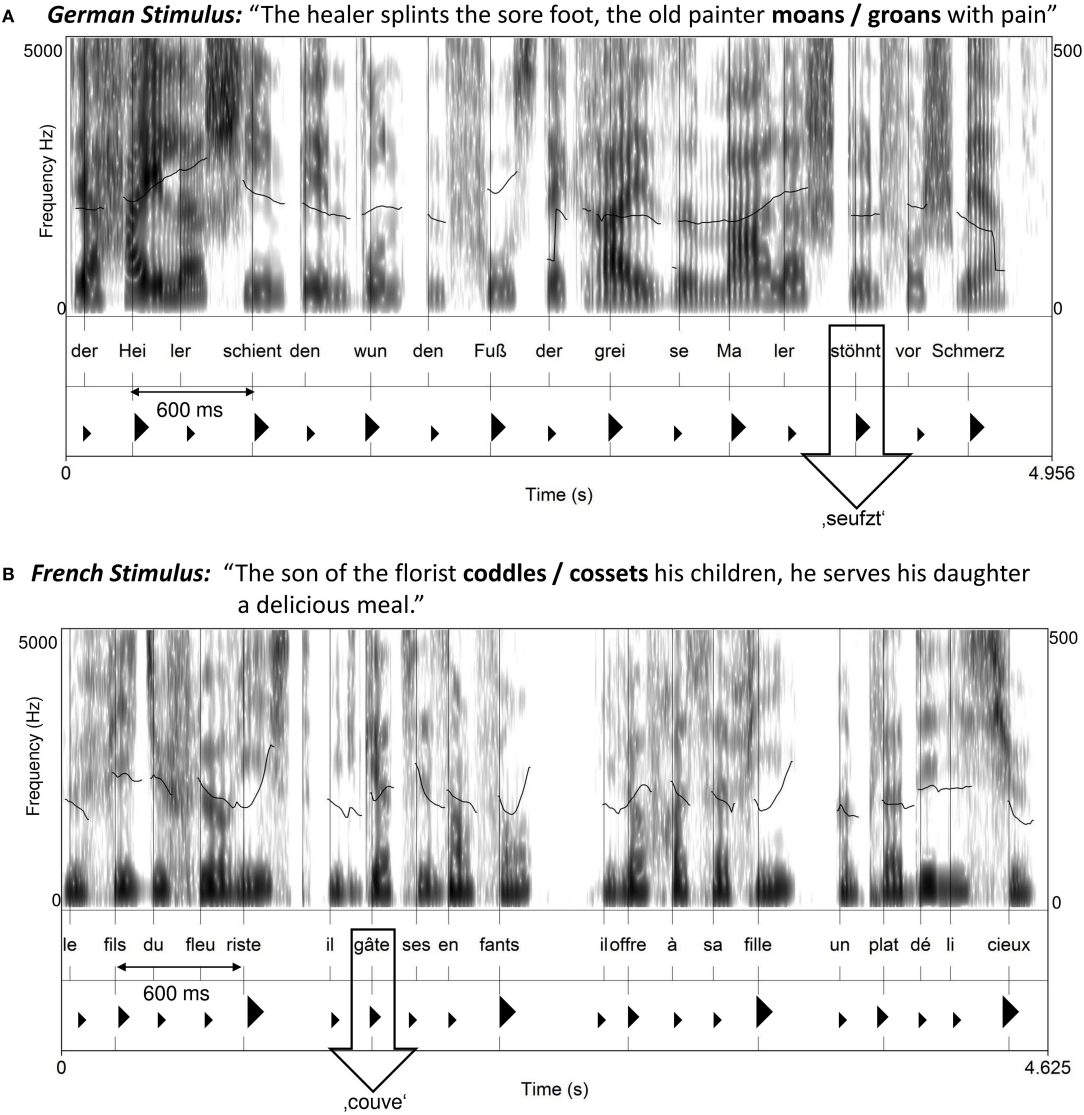
**Examples of (A)** German and **(B)** French stimuli. The bold lines in the spectrogram display the F0 contour. The position of the change verb is indicated by the arrow. In the German example, big triangles mark stressed syllables, small triangles unstressed syllables. In the French example, big triangles mark the main final accents, medium-sized triangles the initial accents, and small triangles, the unaccented syllables.

Twenty-four German and 24 French metrical speech stimuli with regular distributions of accented and unaccented syllables were created (examples in Supplemental Materials). German stimuli (Figure 1A) consisted each of 16 syllables with an alternating binary strong-weak (i.e., stressed–unstressed syllables) metrical pattern. Syntactically, each stimulus was formed by two short simple sentences (8 syllables each, only main clauses) one of which contained the target word (i.e., a monosyllabic verb) that served to test change detection. The occurrence of the verb in the first or in the second sentence was equally balanced across the stimuli. The verb was always placed on a metrically strong position. French stimuli (Figure 1B) consisted of 20 syllables each, divided into four accentual phrases (AP) of five syllables. Each accentual phrase followed a LHLH pattern (Welby, 2007) with an initial and a final accent on the second and on the fifth syllable, respectively. Syntactically, as done with German material, each stimulus included two short simple sentences formed by two accentual phrases each. Across the stimuli, the first and the second sentence contained equally often the monosyllabic target verb. In French, the verb always occurred on an initial accent position.

**Figure 2:**
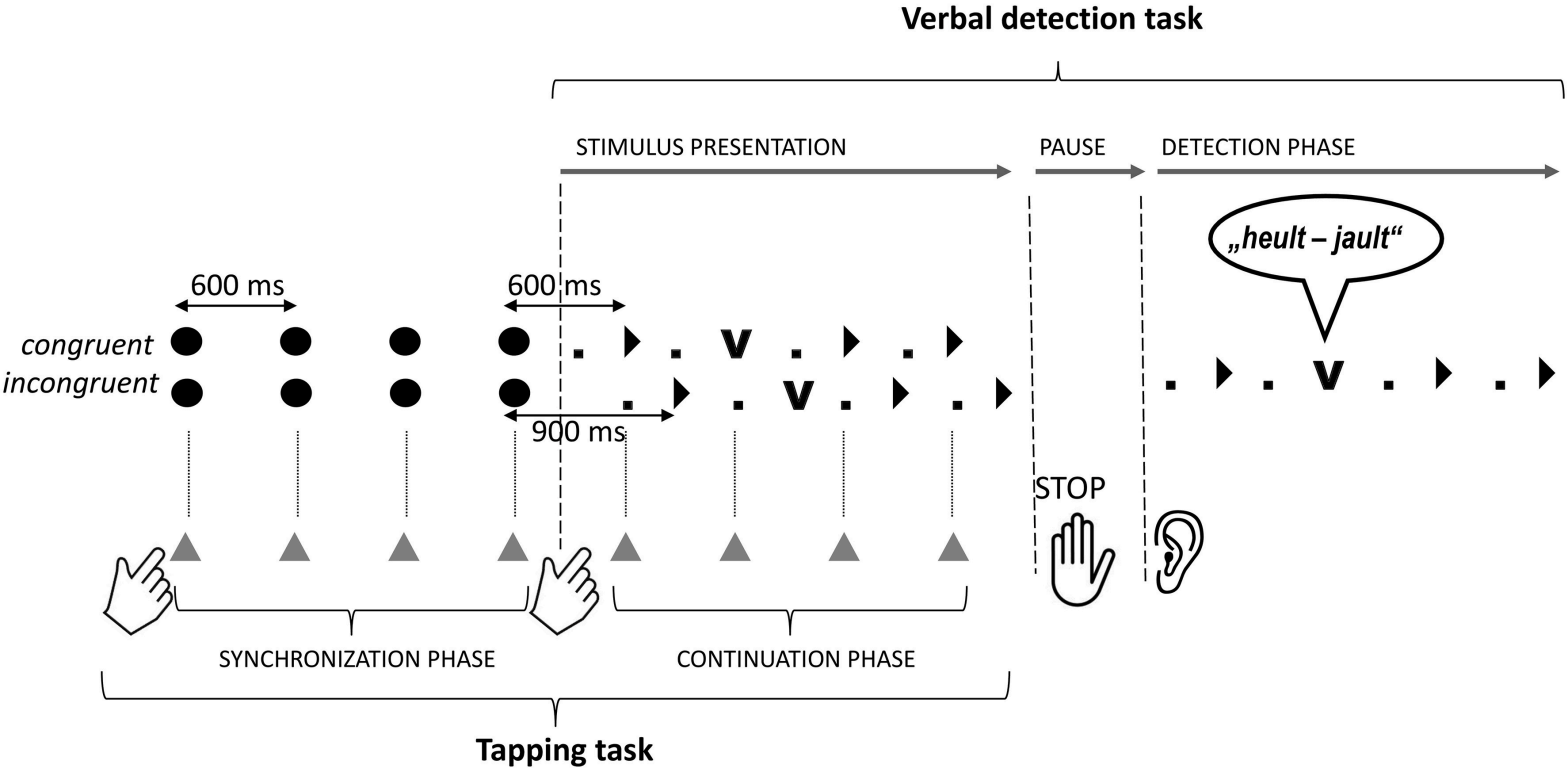
**Procedure used in the Experiment**. A trial (German) is displayed. Accented positions of the metrical speech pattern are marked by black triangles and unaccented positions by small dots. The position of the verb change is marked by a big “V.” The alignment cue (big black dots) and alignment of finger taps (gray triangles) with the speech are displayed.

The stimuli were recorded by two native French- or German-speaking female speakers reading at a regular pace (100 beats/min). The speakers were cued by a metronome prior to reading each stimulus to produce an accented syllable at regular temporal intervals, every 600 ms. The recordings were then examined to ensure that accented syllables occurred every 600 ms, on average (±20 ms). To this aim, the perceptual centers (i.e., p-centers) of accented syllables were estimated using an automated procedure proposed by Cummins and Port (1998). A p-center is defined as the time at which the occurrence of a syllable is perceived, which is most often around the vowel onset (Morton et al., 1976). It can be roughly estimated as the point corresponding to half of the amplitude rise before reaching maximum amplitude for a nuclear vowel (Cummins and Port, 1998). The intervals between the estimated p-centers of accented syllables were calculated, and, if deviating from the 600 ms interval, they were manually adjusted by slightly shortening or lengthening silences or segmental material in the interval, using Praat software (Boersma, 2001). A trial was created in which a stimulus was repeated with a 2-s pause between presentations. In the second presentation (“detection phase”), the target verb was replaced by another verb that had the same morpho-syntactic structure and very similar meaning (e.g., *jault* “yowls”—*heult* “howls”; Figure 2, Sturt et al., 2004).

Semantic closeness between the change verbs and the original verbs (on a scale from 1 = very distant to 10 = very close in meaning) was confirmed in a pilot experiment with two groups of 10 native French and German speakers each (see Table 1). Both verbs were comparable in number of phonemes and frequency (Table 1). For each language, additional filler trials were created to ensure that participants did not pay selectively attention to the verbs. Fillers had the same metrical and syntactic structure as described above. In 24 fillers, a noun was changed, and in 12 fillers, there was no change. Overall, there were 60 trials, including experimental stimuli and fillers, per language.

**Table 1:** **Characteristics of verb changes for French and German material**.

**Language (Database)**	**German material (Dlexdb)**		**French material (Lexique-3)**	
	**Original verb Mean (*SD*)**	**Changed verb Mean (*SD*)**	**Original verb Mean (*SD*)**	**Changed verb Mean (*SD*)**
Number of phonemes	4.1 (0.58)	4.3 (0.38)	4.5 (2.0)	4.3 (1.1)
Frequency	11 per million (28)	16 per million (49)	58 per million (85)	41 per million (56)
Semantic distance rating (1–10)	7.62 (1.25) “semantically close meaning”		7.71 (1.02) “semantically close meaning”	

### Procedure

As described in Falk and Dalla Bella (2016), participants performed a rhythmic finger tapping task (i.e., synchronization-continuation task; Wing, 2002) combined with a verbal change detection task (Sturt et al., 2004; for an illustration, see Figure 2). Prior to each verbal stimulus, participants were asked to tap with the index finger of their dominant hand to 12 isochronous metronome tones (*synchronization phase*; tone duration = 30 ms, Inter-Onset-Interval, IOI = 600 ms). The time of their taps was recorded on the left panel of a Roland SPD-6 MIDI percussion pad. When the metronome stopped, a speech stimulus started. Participants were instructed to continue their tapping at the rate previously indicated by the metronome (*continuation phase*), while carefully listening to the verbal stimulus. The continuation of the taps either resulted in congruent or incongruent alignment of the motor rhythm with the verbal rhythm, depending on the onset of the verbal stimulus. When the alignment condition was *congruent*, the first metrically strong syllable (i.e., its p-center) started 600 ms after the last metronome tone, corresponding to one IOI of the metronome. Thus, participants who continued tapping at the pace of the metronome, aligned their taps to the accented syllables of the speech stimulus. Their taps also occurred on the syllable that contained the change verb. In contrast, when the alignment condition was *incongruent*, the speech stimulus started with a delay of 300 ms, as compared to the congruent condition (Figure 2). As a result, participants' taps continuing at the pace of the metronome fell between accented syllables and did not coincide with the target verb. During the pause preceding the detection phase, participants stopped tapping and prepared for detecting a verbal change. When perceiving a change, they tapped as fast as possible on the right panel of the percussion pad, thereby stopping the stimulus. At the end of the trial, participants recalled the original and the changed word. Verbal answers were recorded with a head-mounted microphone and written down by the Experimenter. In addition, participants summarized the content of the stimuli every three trials on average to ensure that meaning was processed.

Participants were only tested on the stimulus set of their native language. A block of 30 trials (12 stimuli, 12 fillers, and 6 no-change stimuli) per alignment condition was preceded by three practice trials. The stimulus set for each language was organized in 8 randomization lists which were presented equally often under both alignment conditions in counterbalanced order across participants.

A perceptual Baseline condition, without cues and tapping for the French and German stimuli was additionally tested with two other groups of participants in order to evaluate motor effects on detection sensitivity (see Falk and Dalla Bella, 2016). The verbal change detection procedure followed the same protocol and randomization procedure as described above. However, no metronome cue was presented before the stimuli and participants only listened to the stimuli before giving their verbal response.

The tapping Experiment lasted approximately 45 min, and the Baseline assessment 35 min. Both were run on an IBM-compatible computer using MaxMSP 5.1.9. Auditory stimuli were presented via Beyer-Dynamic DT-770 Pro 250 headphones (German group) and Beyer-Dynamic MMX 300 headphones (French group). Testing was done in Munich, Germany, and Aix-en-Provence, France.

### Tapping analyses^1^

#### General tapping measures

Tapping rate was obtained by calculating the mean inter-tap-interval (mean ITI). The expected mean ITI was 600 ms, as cued by the metronome, in both the synchronization and continuation phases. Motor variability was obtained by computing the Coefficient of Variation of the ITI (CV ITI), namely the standard deviation of the ITI divided by the mean ITI. Higher CV indicates higher motor variability during the task.

#### Synchronization phase

We examined whether participants' taps were well-aligned with the metronome tones. To this goal, synchronization accuracy (i.e., the synchronization error and its direction) and synchronization consistency were calculated (e.g., Aschersleben, 2002; Repp and Penel, 2004; Repp, 2005; Sowiński and Dalla Bella, 2013; Dalla Bella et al., 2016). The synchronization error and its direction were obtained by computing the absolute and the signed (positive or negative) mean delays between the taps and the metronome, respectively. Negative direction of the synchronization error indicates that the taps precede the metronome tone, on average. Synchronization consistency is measured by the standard error (*SE*) of the asynchrony between taps and metronome tones.

#### Continuation phase

Performance in the continuation phase was measured to assess how well the participants followed the instruction to continue tapping at the pace indicated by the metronome. Similar to the measures in the synchronization phase, measures of continuation accuracy and consistency were obtained, in spite of the absence of an explicit pacing stimulus. For accuracy, we measured whether the taps occurred on average before or after the *expected* tap times as indicated by the preceding metronome. Thus, mean signed asynchrony (positive or negative) was calculated between the actual tap times and the *expected* tap times from the synchronization phase. Consistency in the continuation phase was determined as the *SE* of the asynchrony between the actual taps and the *expected* tap times.

## Results

### Detection performance

Verbal responses in the detection phase were analyzed by calculating sensitivity (*d*′) and response bias (*C*, MacMillan and Creelman, 2005) as done in our previous study (Falk and Dalla Bella, 2016). A Hit occurred when both the changed and the original verb were provided in their semantically and phonetically accurate form. A False alarm occurred when a change was reported in a no-change trial. Data of one French-speaking participant in the main Experiment were discarded because she performed at chance in the detection task (*d*′ = 0).

**Figure 3:**
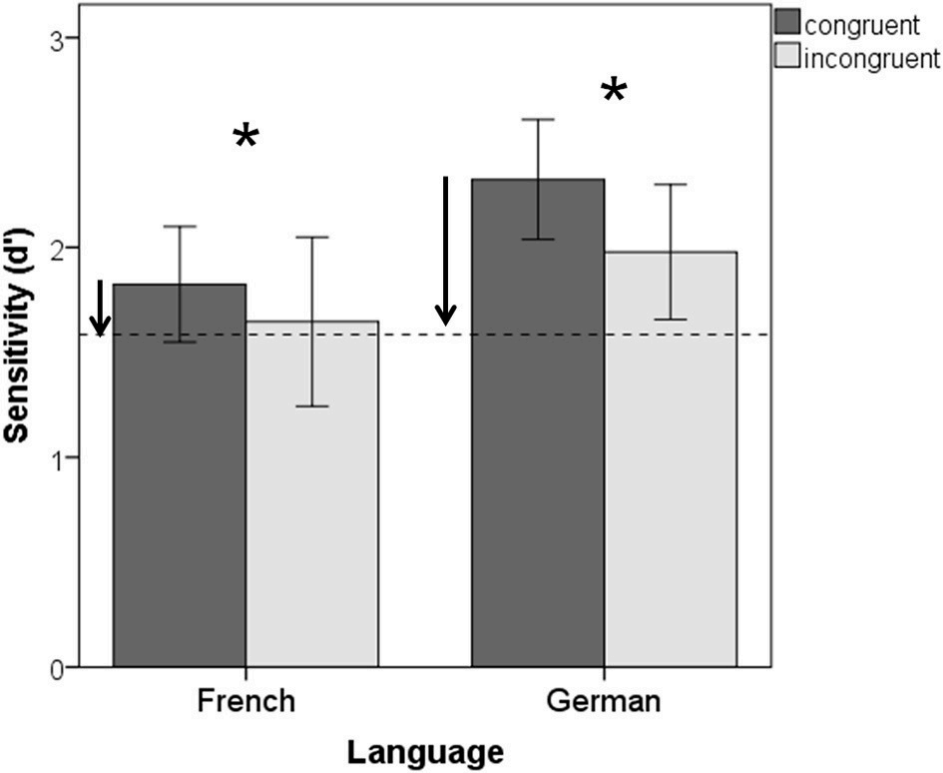
**Detection sensitivity for change words in congruent and incongruent tapping conditions for French and German speakers**. The perceptual baseline (averaged across language groups) is displayed as a dotted line. Error bars represent 95%-Confidence intervals. Stars indicate significant differences between alignment conditions. Arrows indicate significant differences between the Baseline and the motor alignment conditions.

We first examined if detection of test words (*d*′) was influenced by motor alignment in the tapping task. Data were entered in a 2 × 2 mixed-design Analysis of Variance (ANOVA) taking Congruency (congruent vs. incongruent) as the within-subject factor, and Language (French vs. German) as the between-subject factor. As shown in Figure 3, regardless of language, participants were overall more efficient in detecting verbal changes when their taps were congruently aligned to speech accents, as compared to the incongruent alignment [*F*_(1, 61)_ = 4.024, *p* = 0.049]. Overall, greater sensitivity to change detection was found in the German than in the French-speaking group^2^ [*F*_(1, 61)_ = 5.15, *p* = 0.027]. The Congruency × Language interaction did not reach significance. Response bias (*C*) was entered into a similar ANOVA. No differences in response bias were found, as indicated by lack of significant effects of Congruency, Language, and their interaction.

Second, we compared the tapping data in the congruent and incongruent conditions taken separately to the perceptual Baseline data (French: *d*′ = 1.51, 95%-CI = ±0.469; German: 1.65, 95%-CI = ±0.469). Two 2 × 2 ANOVAs were run using Language (French vs. German) and Condition (Motor alignment vs. Baseline) as between-subject factors. Results revealed that the congruent motor alignment enhanced detection sensitivity relative to the Baseline [*F*_(1, 91)_ = 8.16, *p* = 0.005] in both languages (no main effect of Language, no interaction). No difference in sensitivity was found between the incongruent alignment and the Baseline (*p* > 0.27).

### Tapping performance^3^

#### General motor performance

To assess motor performance, tapping rate (mean ITI) and motor variability (CV of ITI) in the synchronization and continuation phases were analyzed for all trials. Data were entered in two independent 2 × 2 × 2 mixed-design ANOVAs. Congruency (congruent vs. incongruent) and Phase (synchronization vs. continuation) were the within-subject factors, while Language (French vs. German) was the between-subject factor.

**Figure 4:**
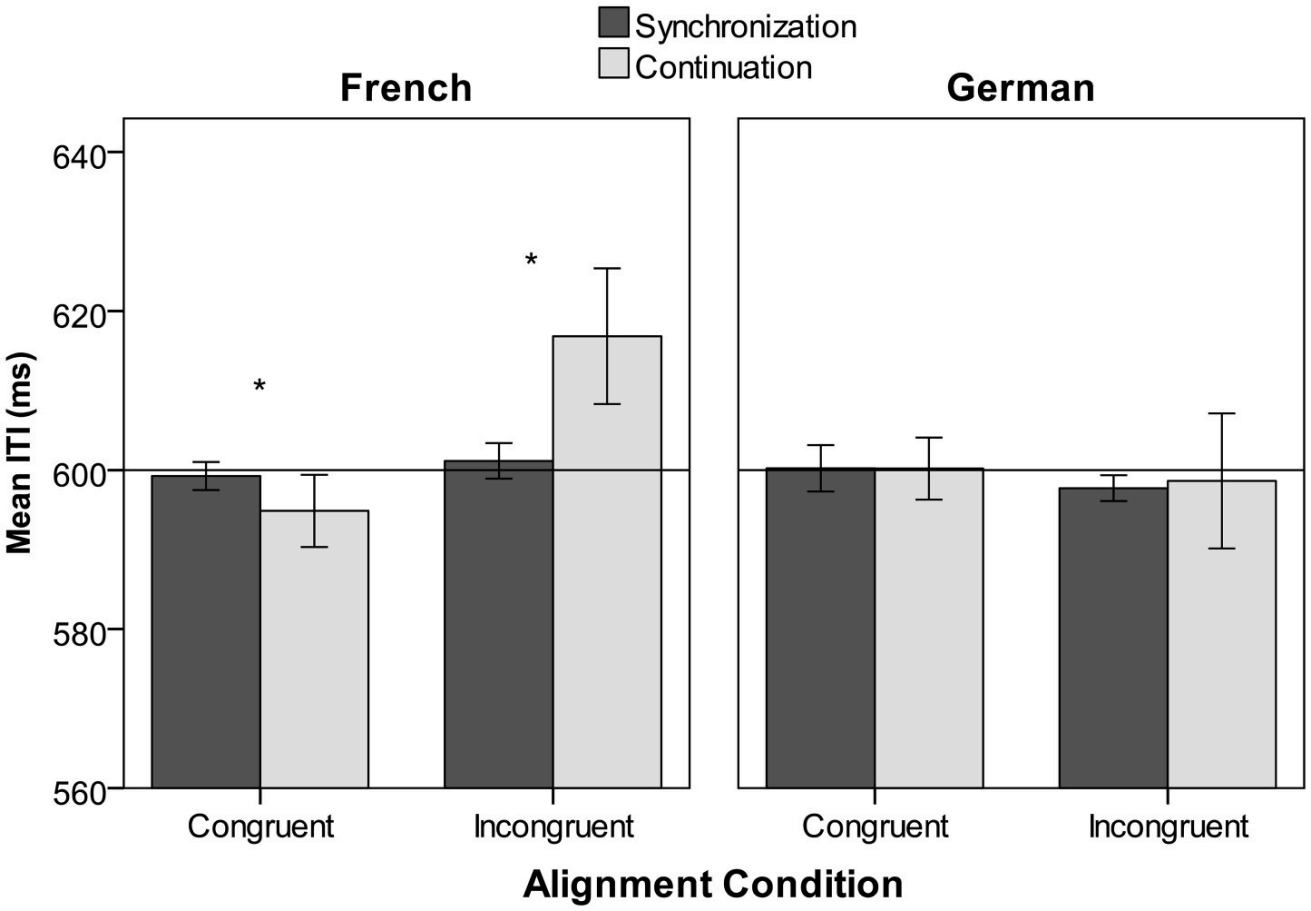
**Tapping rate (mean ITI) in the synchronization phase (with metronome) and the continuation phase (with speech) in the two alignment conditions, for French and German speakers**. The horizontal line indicates the expected ITI at 600 ms. Error bars represent 95%-Confidence intervals. Stars indicate significant differences.

For tapping rate, the triple interaction Congruency × Language × Phase was highly significant [*F*_(1, 61)_ = 16.15, *p* < 0.001]. This interaction was decomposed by holding Language constant, and by running two additional 2 × 2 ANOVAs. As can be seen in Figure 4 (right panel), German participants tapped in the vicinity of the expected interval (mean ITI = 599 ms, 95 %-CI = ±3 ms) without showing any differences between the synchronization and continuation phase, nor between congruent and incongruent alignments. In contrast, in the French group, analyses revealed a significant Congruency × Phase interaction [*F*_(1, 30)_ = 32.96, *p* < 0.001; main effect of Congruency: *F*_(1, 30)_ = 42.37, *p* < 0.001; main effect of Phase: *F*_(1, 30)_ = 4.58, *p* = 0.041]. French-speaking participants (Figure 4, left panel) slightly accelerated when tapping with speech as compared to the metronome in the congruent condition [*F*_(1, 30)_ = 4.93, *p* = 0.034], but slowed down on average by ~15 ms per ITI when tapping to speech in the incongruent condition [*F*_(1, 30)_ = 11.20, *p* = 0.001].

The two language groups did not differ in terms of motor variability (CV ITI_French_ = 0.054, CI = ±0.005; CV ITI_German_ = 0.053, CI = ±0.005). Overall, participants' motor variability only differed in the continuation phase depending on the congruency condition [Congruency × Phase interaction: *F*_(1, 61)_ = 8.91, *p* = 0.004; main effects for Congruency: *F*_(1, 61)_ = 6.02, *p* = 0.017; and Phase: *F*_(1, 61)_ = 70.00, *p* < 0.001]. Participants' motor variability was higher when tapping incongruently to speech in the continuation phase than when tapping congruently [CV_con_ = 0.048, CI = ±0.004 vs. CV_inc_ = 0.064, CI = ±0.005, *F*_(1, 62)_ = 11.35, *p* = 0.001].

#### Tapping in the synchronization phase as a predictor of detection performance

An additional goal of the present study was to examine whether synchronization with the metronome tones was a predictor of participants' success in later detection performance. We tested first if the two language groups differed in overall synchronization skills across all trials. Synchronization accuracy (synchronization error and its direction) and consistency (*SE* of asynchrony) were entered into three separate 2 × 2 mixed-design ANOVAs, with Congruency as the within-subject factor and Language as the between-subject factor. Overall, the results showed good synchronization with no differences between language groups or congruency conditions. All the participants tapped with a synchronization error of 57.44 ms on average (95%-CI = ±6.72 ms). Their taps rather preceded the metronome tones (synchronization direction = −53.15 ms; 95%-CI = ±7.52 ms), as typically reported in tapping studies (negative mean asynchrony, e.g., Aschersleben, 2002). Synchronization consistency was within the normal range (mean *SE* = 9.05; 95%-CI = ±0.5) for a comparable population of young adults (e.g., Dalla Bella et al., 2016).

After having discarded the possibility of group differences in synchronization skills, we examined whether participants' synchronization performance across language groups could predict sensitivity in change detection (*d*′) in the congruent and incongruent alignment conditions, respectively. To this aim, we fitted linear regression models in which *d*′ was the dependent variable and the predictors were Language and the variables reflecting the tapping performance during the synchronization phase: synchronization accuracy (i.e., signed asynchrony), synchronization consistency, tapping rate and motor variability. The best-fitting model is reported below (Table 2)^4^. In the congruent condition, *d*′ was predicted by the direction of synchronization error: the more the participants tapped in advance of the metronome tones in the synchronization phase (i.e., the more negative their signed asynchrony), the higher the *d*′ for both language groups in the continuation phase. This model was not a good fit to the data from the incongruent condition (*p* > 0.60).

**Table 2:** **Predictors of sensitivity in change detection (*d*′) in the congruent alignment condition**.

	**B/Beta**	**Std. Error**	***t***	***p***
Constant	1.455	0.217	6.690	<0.001^**^
Language	0.578/0.362	0.192	3.004	0.004^**^
Signed asynchrony	−0.006/−0.259	0.003	−2.151	0.036^*^

*Model: R^2^ = 0.16, F_(2, 60)_ = 5.81, p = 0.005, AIC = 147.41, BIC = 155.98*.

* *p < 0.05*;

** *p < 0.01*.

#### Tapping in the continuation phase as a predictor of detection performance

**Figure 5:**
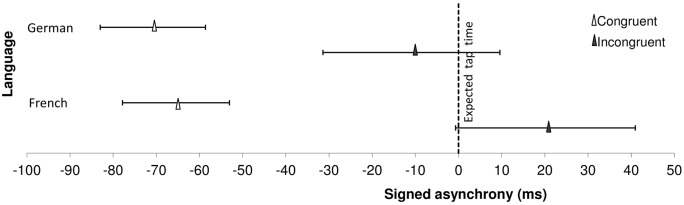
**Continuation accuracy (signed asynchrony) for French and German speakers in relation to the expected moment of the tap (dotted vertical line at 0 ms) during the continuation phase (i.e., with speech) depending on the alignment condition**. Triangles represent the mean asynchrony, error bars display 95%-Confidence intervals.

Here, we examined whether tapping performance in the continuation phase predicted participants' detection performance. As before, we tested potential differences in the tapping task, in terms of accuracy and consistency during continuation in the two language groups with two 2 (Congruency) × 2 (Language) repeated-measures ANOVAs. Figure 5 shows the results for accuracy (signed asynchrony) for the two language groups. As can be seen, all the participants tapped in advance relative to the expected tap time in the congruent condition compared to the incongruent condition [main effect of Congruency, *F*_(1, 61)_ = 96.85, *p* < 0.001]. However, continuation accuracy differed between French and German speakers as a function of the alignment condition [Congruency × Language interaction, *F*_(1, 61)_ = 4.67, *p* = 0.035]. With incongruent alignment, the French-speaking participants tapped later (i.e., after the expected tap time) than the German-speaking participants who were still slightly in advance of the expected tap time [*F*_(1, 61)_ = 4.52, *p* = 0.038]. All participants, irrespective of language group, were less consistent when their taps were incongruently aligned with the speech accents than when they were congruently aligned [*F*_(1, 61)_ = 96.85, *p* < 0.001]. No differences in consistency were found between language groups nor interactions with the alignment condition (French group, mean consistency = 23.69, CI = ±3.20; German group, mean consistency = 21.81, CI = ±3.15).

**Figure 6:**
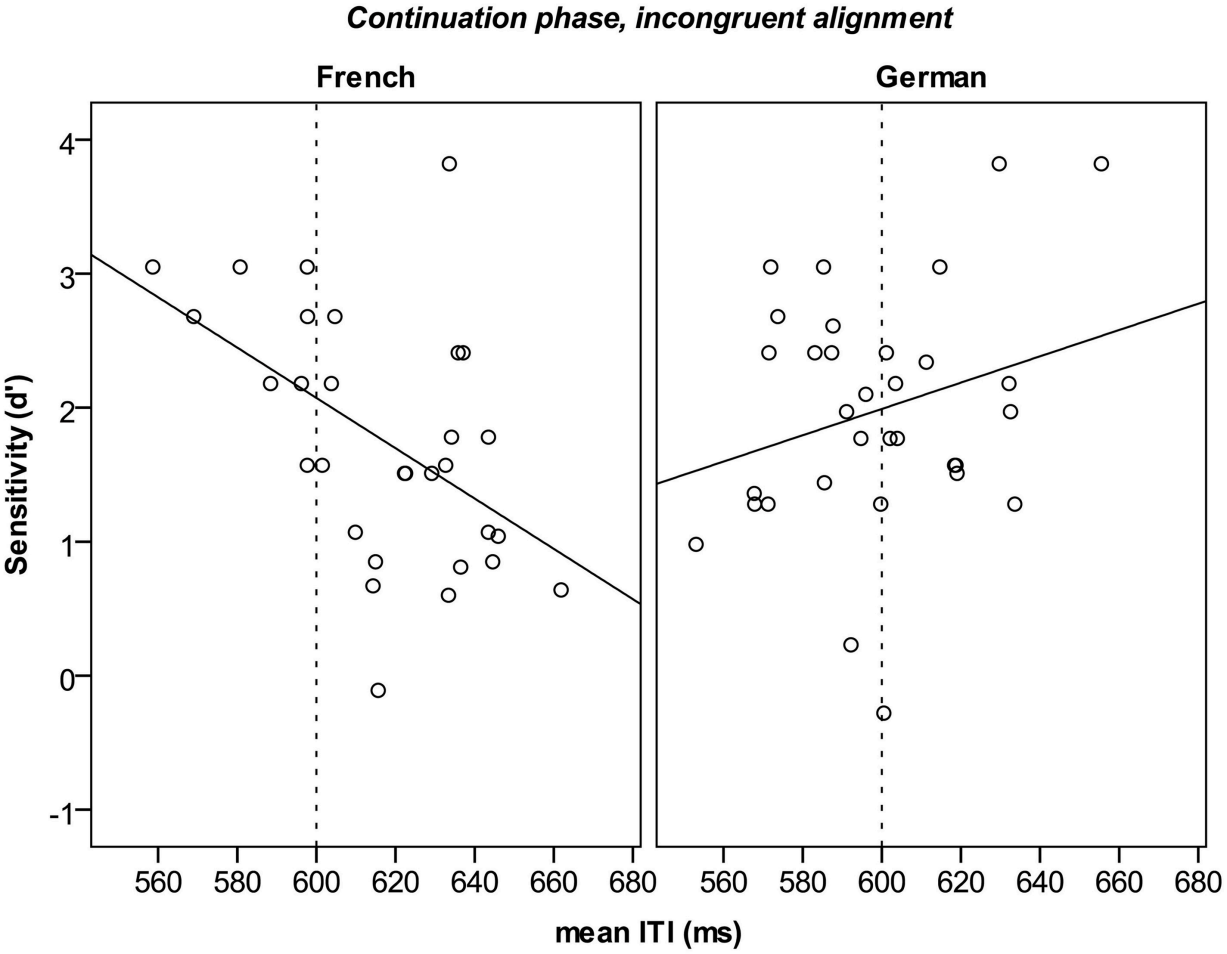
**Individual detection performance (*d*′) in relation to tapping rate (mean ITI) in the continuation phase/incongruent alignment by language group**. The regression lines are displayed in bold. The dotted lines mark the expected ITI.

Participants' tapping performance in the continuation phase for test trials was used to predict detection success (*d*′). We fitted linear regression models in which the dependent variable was sensitivity (*d*′) and the predictors were language, continuation accuracy, consistency as well as tapping rate and motor variability. Again, the best-fitting model is reported^4^. Tapping in the continuation phase predicted detection performance only in the incongruent condition. The best model (Table 3) showed that the tapping rate was a significant predictor for *d*′, and that the relation between *d*′ and tapping rate differed with respect to language, as can be seen in Figure 6.

**Table 3:** **Predictors of sensitivity in change detection (*d*′) in the incongruent alignment condition**.

	**B/Beta**	**Std. Error**	***T***	***p***
Constant	13.336	3.951	3.375	0.001^**^
Language	−17.238/−9.586	5.530	−3.117	0.003^**^
Tapping rate	−0.019/−0.529	0.006	−2.933	0.005^**^
Language^*^Tapping rate	0.029/0.016	0.009	3.145	0.003^**^

*Model: R^2^ = 0.17, F_(3, 58)_ = 4.00, p = 0.01, AIC = 161.06, BIC = 171.70*.

** *p < 0.01*.

**Figure 7:**
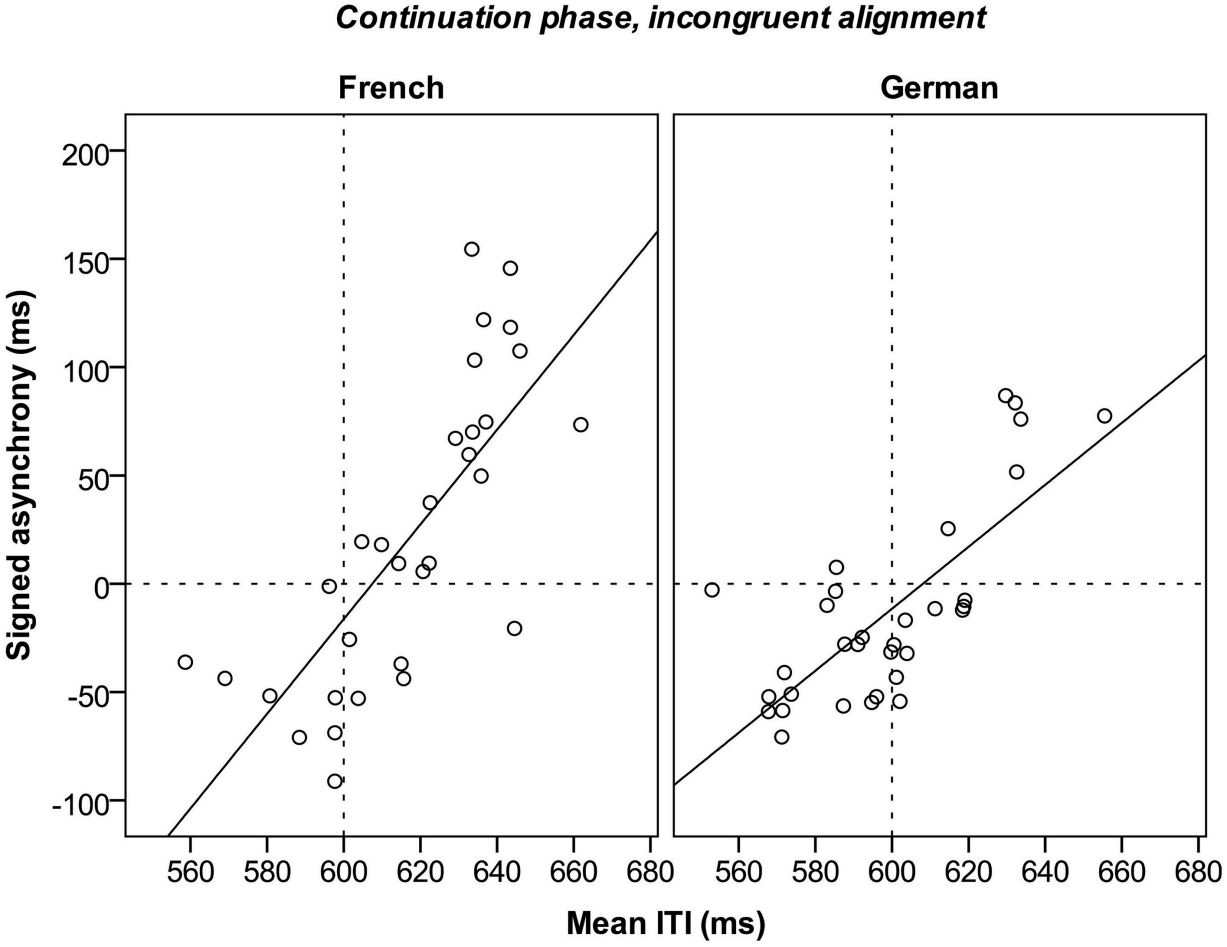
**Continuation accuracy (signed asynchrony) in relation to tapping rate (mean ITI) in the continuation phase (incongruent alignment) for the two language groups**. The regression lines are displayed in bold. The dotted lines mark the expected moment of tap on the y-axis and the expected tapping rate (continued from the synchronization phase) on the x-axis.

In light of these results, we further examined whether there was a relation between tapping rate and continuation accuracy in test trials for the incongruent alignment condition. Note that there are overall differences in continuation accuracy and also tapping rate between language groups, as observed before (see Figures 4, 5). Simple linear regressions were performed separately for each language group with tapping rate as the predictor and continuation accuracy as the dependent variable. Tapping rate accounted for a high proportion of variance in continuation accuracy for the incongruent alignment for both French [*R*^2^ = 0.58, *F*_(1, 29)_ = 39.2, *p* < 0.001] and German [*R*^2^ = 0.56, *F*_(1, 30)_ = 38.8, *p* < 0.001] participants, as shown in Figure 7. Thus, participants tapping at a slower rate than expected were also those who lagged more behind the expected moment of the tap in the incongruent continuation phase.

## Discussion

The aim of the present study was to test whether the beneficial effects of aligning a motor rhythm to a speech rhythm on verbal processing are found in rhythmically diverse languages (i.e., French, a non-stress language vs. German, a lexical stress language). In addition, we investigated the links between individual motor performance and benefits in speech processing. French and German native speakers were cued to tap their finger with metrical speech stimuli they heard in their native language. Tapping was either aligned with the speech accents (congruently) or non-aligned (incongruently; see Falk and Dalla Bella, 2016). After tapping, participants detected subtle verbal changes in the speech stimuli.

Our results showed that native speakers of both a lexical stress- and a non-stress language were more successful in detecting subtle verbal changes in speech, when their finger taps were congruently aligned with the accented syllables of the sentences compared to incongruent alignment and to a perceptual baseline. Thus, the benefits of aligning motor and speech rhythms on verbal processing, already shown in German (Falk and Dalla Bella, 2016), extend to a non-stress language (French). Generalization of the effect to rhythmically different languages supports the idea that motor alignment with prominent syllables in speech enhances attending to expected moments in time (e.g., Chemin et al., 2014), and thereby verbal processing. Although French and German accentuation substantially differs in prosodic embedding, function, and acoustic correlates, the present findings support the idea that both types of rhythmic organization similarly serve as anchors for linking perception and action in time through shared predictions in a metrical context. This is in line with previous results pointing to a close link between speech prominences and manual movements (Kelso et al., 1985; Rochet-Capellan et al., 2008; Parrell et al., 2014). Yet, note that the present study did not allow us to directly compare sensitivity in French vs. German listeners, as the material in the two languages was very different in terms of semantics, syntax, and phonetics/phonology.

We further investigated which aspects of the motor performance can predict individual success in the verbal task. We identified two motor predictors for verbal detection, synchronization performance with the cue and tapping rate during continuation, which differed depending on the alignments between motor and auditory rhythms. First, when rhythms were aligned (congruent condition), participants who tapped earlier to the metronome tones preceding the verbal stimulus had more success in detecting a verbal change than the other participants. Tapping in advance of a discrete, periodically recurring and, thereby, *expected* sound event is a common finding in tapping research (negative mean asynchrony; Fraisse and Voillaume, 1971; Aschersleben, 2002; Repp and Su, 2013). It is indicative of anticipatory behavior during synchronization (Repp, 2005; van der Steen and Keller, 2013). The possibility that expectancies emerging during the synchronization phase can influence subsequent speech processing is supported by brain research on auditory cueing with speech (e.g., Cason and Schön, 2012). In a recent perceptual EEG-study with the same French material (Falk et al., in press), sentences were cued by an auditory cue matching or non-matching the rhythm of speech (no motor condition). Akin to the present study, the authors found that participants' success in behavioral response (a word memory task) was correlated with neural activity during the cueing but not during the speech phase. In particular, participants whose neural oscillatory activity showed better phase-locking (i.e., “neural entrainment,” Lakatos et al., 2008) to the rhythm of the cue, also had higher success in the following speech memory task. Neural entrainment has been described as a process that drives neural excitability toward expected times and thereby engages higher attentional resources at these points in time (e.g., Calderone et al., 2014). This neural effect on attending is even more pronounced when motor synchronization is added (e.g., Morillon et al., 2014). Therefore, in our experiment, participants generating stronger expectancies during the synchronization phase may also be those that are more likely to maintain these expectancies during the continuation phase and having higher attentional resources to allocate. Thus, they may be more prone to get cognitive and neural benefits from rhythmically induced attending to speech when temporal expectancies are met by the speech structure (i.e., in the congruent condition). By contrast, in the incongruent condition, expectancies from the synchronization phase cannot be used to enhance attending because of the subsequent misalignment of taps and speech accent structure. The misalignment may disrupt expectancies directed to the relevant verbs and, hence, no relation between the synchronization phase and detection performance is found. Finally, it is interesting that we failed to observe a relation between continuation accuracy and verbal detection in the congruent condition. This may be linked to additional processes intervening during speech processing (e.g., comprehension, syntactic, or semantic processes) or to task-related factors (e.g., participants were not explicitly instructed to synchronize with stressed syllables while listening to speech). These possibilities deserve further investigation in future studies.

Second, when rhythms were not aligned (incongruent condition), tapping rate in the continuation phase (i.e., while participants listened to speech) predicted detection of a verb change, but differently depending on the language. Slowing down tapping in the continuation phase worsened detection in French speakers, while this was not the case in German speakers. This group difference may be linked to the fact that French speakers generally showed a considerable drift in tapping rate (a deceleration of 15 ms per ITI, on average) in the incongruent alignment condition which naturally also affected continuation accuracy. German speakers did not show any drift. Moreover, slower tapping rate in the continuation phase was associated with more positive asynchrony (i.e., lag) relative to the expected moments of the tap. In sum, French speakers, unlike German speakers, tended to lag behind the expected moment of the taps when motor and speech rhythms were incongruently aligned and this drift was linked to worse detection performance. In the following, we will discuss different explanations that can account for these findings.

A relatively trivial explanation is that pre-existing differences in musical experience between language groups may have led to worse tapping performance. However, musical experience should have produced group differences in all aspects of tapping performance, which was not the case (e.g., in the synchronization phase). Moreover, including musical experience as an additional predictor in the regression models did not change the pattern of results. Hence, differences in musical experience are unlikely to account for the observed group differences.

A more interesting explanation is that a dual task (i.e., tapping + listening to speech) during the continuation phase affected French and German speakers differently in our experiment. Generally, as already shown in Falk and Dalla Bella (2016), dual task conditions when auditory and motor rhythms are aligned lead to *enhanced* detection performance compared to a single task perceptual baseline. This finding indicates efficient integration of motor with speech accent information when both are temporally aligned. Similar beneficial multisensory effects of manual gesturing were reported in speech perception (Holle et al., 2012; Biau and Soto-Faraco, 2013; Ito et al., 2014). In contrast, incongruent alignment, by temporally dissociating prominent verbal and motor information, creates less ideal conditions for efficient auditory-motor integration. Although, a stable rhythmic relation may still be established between speech and motor rhythms when finger taps are cued to occur at the anti-phase between accents (see Vos and Helsper, 1992; Volman and Geuze, 2000; Repp, 2005), more difficulties in maintaining a stable relation may be encountered by participants. In our study, both higher motor variability in the continuation phase and lower continuation consistency were indicators of increased difficulty (or lower stability) in this condition. On the other hand, it appears that destabilizing the speech-motor relation in the incongruent alignment condition showed interesting differences between language groups, possibly because listeners had to readjust their coordination with the auditory stimulus. An important difference was the remarkable tapping deceleration found in French, but not in German speakers. One possibility is that, in the German material, the alternating strong-weak pattern made it easier for listeners to track strong syllables as reference points when tapping on the weak syllable between them. In the French material, the ternary pattern may have made it more difficult to find the anchor for tapping between two metrically strong syllables. However, there is another explanation that takes accentual properties of the two languages into account. Previous studies reported that a concurrent speech or musical rhythm (distractor sequence) makes listeners deviate from a synchronized tapping pattern to a metronome (target sequence; Dalla Bella et al., 2013). In this kind of task, a lagging distractor rhythm typically leads to positive asynchronies in concurrent synchronization performance (Repp, 2003; Repp and Penel, 2004). Similarly, positive asynchronies were observed in our experiment during incongruent continuation tapping, particularly for the French speakers that also showed significant deceleration. This suggests that properties inherent to the language-specific metrical pattern may have attracted the French speakers more to the upcoming speech accent(s) than the German speakers. Final accents (right-edge of the AP) could have played a major role in this process. Indeed, these final accents feature higher and steeper rises and vocalic lengthening and appear to be more prominent than initial accents (left-edge of the AP, e.g., Rolland and Lœvenbruck, 2002). In addition, in corpora of colloquial French, initial accents are more optional and seem to depend on stylistic variation in contrast to the obligatory and highly predictable phrase-final accents (e.g., Astésano, 2001). This imbalance in accentual acoustics and predictability may have led French speakers to perceive more prominently the periodicity of primary (i.e., final) accents occurring at the right edges of the AP in our study. These accents occurred at a periodicity of 1,200 ms, and thereby, on a higher metrical level compared to the 600-ms periodicity of both initial and final accents. Note that, in a previous study, French speakers were also more likely to spontaneously tap to a higher periodicity in metrical speech compared to English speakers, although it was not possible to identify the specific linguistic landmarks attracting the tapping in this study (Lidji et al., 2011). In sum, tapping deceleration for French speakers observed in the incongruent condition may have been driven by attending to the higher metrical level of final accents (i.e., to a higher periodicity). Moreover, as the verbal changes exclusively occurred on initial accents, participants with higher attraction to final accents may have displayed worse detection performance. Future studies, placing verbal changes in final and initial AP positions, will help to examine this possibility. Alternatively, including languages with different accentual properties in a future study (such as Spanish, which possesses lexical stress, but different acoustic and positional properties than French and German, e.g., Toro et al., 2009) may further clarify the potential of motor alignment tasks to inform us about the perception and adaption to accentual salience.

Finally, it is noteworthy that the congruent alignment condition did not produce a similar drift in French speakers. Previous synchronization studies using finger tapping have shown that *1:n* metrical subdivisions are generally not deleterious to synchronization capacity, rather the contrary, provided that different periodicities are coupled in a hierarchical system (Large and Jones, 1999; Large and Palmer, 2002; Repp, 2008). Thereby, the congruent condition may have provided a metrical template with clear nesting of temporal reference frames during auditory-motor integration for French speakers even when they were attracted by the higher metrical level.

To conclude, differences in auditory-motor coordination between French and German speakers open interesting perspectives for further investigating differences in accent perception and metrical reference frames for multisensory processes. Overall, our results support recent models of expectancy-driven speech perception underscoring the role of the motor system. Our findings are compatible with Kotz and Schwartze's (2010, 2016) subcortical-cortico framework for speech perception. The model encompasses a few critical regions typically involved in motor control, such as the (pre)supplementary motor area and basal ganglia and cerebellar circuitries. According to the model, the motor timing elements together with the other elements of the neural network converge in their function to optimize predictive timing of verbal behavior. Thereby, the network serves to precisely coordinate speech perception and production in time, and also explains the role of auditory-motor coupling and learning during language acquisition. Thus, motor benefits in speech perception such as found in our study may be the expression of an enhanced stimulation of these underlying neural mechanisms of predictive timing.

Finally, our findings are likely to extend to those ecological settings, whereby metrical speech and auditory-motor interactions are used to foster verbal memory and learning. Children's lore, in particular, exploits metrical speech with synchronized movements (e.g., hand clapping, stamping) in a wide variety of games that enhance children's social and verbal skills (Opie and Opie, 1988). Oratory and joint speech in groups are other domains in which auditory-motor coupling with speech is naturally found which may help to convey verbal messages to a group and improve social cohesion and inter-subjectivity (e.g., Cummins, 2014). Ultimately, our results may encourage novel multisensory rhythm-based interventions that are currently under investigation for fostering language acquisition and learning in developmental populations with speech and language disorders, such as dyslexic children (Schön and Tillmann, 2015) or autistic children (Wan et al., 2011). To conclude, the present results help to advance our understanding of the language-specific bases and learning of synchronized auditory-motor rhythms and predictive timing in multisensory speech processing.

## Author contributions

SF and SDB designed the study. SF and CV developed the material and collected the data. All the authors contributed to data analysis. SF and SDB outlined the article and all the authors contributed to the final manuscript.

### Conflict of interest statement

The authors declare that the research was conducted in the absence of any commercial or financial relationships that could be construed as a potential conflict of interest.

## Footnotes

^1^A MIDI delay of 81 ms was subtracted from all tapping data of the German-speaking group, and of 90 ms for the French-speaking group.

^2^Note that this main effect is not readily interpretable because of the different verbal materials used in the two languages.

^3^Language groups differed in musical experience. Because musical training can affect tapping performance, we re-ran all models reported below showing Language effects by including musical experience as a covariate. Musical experience did not change any of the results.

^4^No differences in the results were found when the regression model was run with and without one extreme value of *d*′ in the incongruent condition in the French group. The results without the extreme value are reported.

## References

[B1] Aschersleben G. (2002). Temporal control of movements in sensorimotor synchronization. Brain Cogn. 48, 66–79. 10.1006/brcg.2001.1304 11812033

[B2] Astésano C. (2001). Rythme et Accentuation en Français. Invariance et Variabilité Stylistique. Paris: Editions L'Harmattan.

[B3] Biau E. Soto-Faraco S. (2013). Beat gestures modulate auditory integration in speech perception. Brain Lang. 124, 143–152. 10.1016/j.bandl.2012.10.008 23333667

[B4] Boersma P. (2001). Praat, a system for doing phonetics by computer. Glot Int. 5, 341–345.

[B5] Bohn K. Knaus J. Wiese R. Domahs U. (2013). The influence of rhythmic (ir)regularities on speech processing: evidence from an ERP study on German phrases. Neuropsychologia 51, 760–771. 10.1016/j.neuropsychologia.2013.01.006 23333869

[B6] Calderone D. J. Lakatos P. Butler P. D. Castellanos F. X. (2014). Entrainment of neural oscillations as a modifiable substrate of attention. Trends Cogn. Sci. 18, 300–309. 10.1016/j.tics.2014.02.005 24630166 PMC4037370

[B7] Cason N. Schön D. (2012). Rhythmic priming enhances the phonological processing of speech. Neuropsychologia 50, 2652–2658. 10.1016/j.neuropsychologia.2012.07.018 22828660

[B8] Chemin B. Mouraux A. Nozaradan S. (2014). Body movement selectively shapes the neural representation of musical rhythms. Psychol. Sci. 25, 2147–2159. 10.1177/0956797614551161 25344346

[B9] Conradi N. Abel C. Frisch S. Kell C. A. Kaiser J. Schmidt-Kassow M. (2016). Actively but not passively synchronized motor activity amplifies predictive timing. Neuroimage 139, 211–217. 10.1016/j.neuroimage.2016.06.033 27329809

[B10] Cummins F. Port R. F. (1998). Rhythmic constraints on stress timing in English. J. Phon. 26, 145–171. 10.1006/jpho.1998.0070

[B11] Cummins F. (2014). Voice, (inter-)subjectivity, and real-time recurrent interaction. Front. Psychol. 5:760. 10.3389/fpsyg.2014.00760 25101028 PMC4102880

[B12] Dalla Bella S. Bialuńska A. Sowiński J. (2013). Why movement is captured by music, but less by speech: role of temporal regularity. PLoS ONE 8:e71945. 10.1371/journal.pone.0071945 23936534 PMC3732235

[B13] Dalla Bella S. Farrugia N. Benoit C.-E. Bégel V. Verga L. Harding E.. (2016). BAASTA: Battery for the Assessment of Auditory Sensorimotor and Timing Abilities. Behav. Res. Methods. [Epub ahead of print]. 10.3758/s13428-016-0773-6 27443353

[B14] Dilley M. C. McAuley J. D. (2008). Distal prosodic context affects word segmentation and lexical processing. J. Mem. Lang. 59, 294–311. 10.1016/j.jml.2008.06.006

[B15] Domahs U. Knaus J. Orzechowska P. Wiese R. (2012). Stress “deafness” in a language with fixed word stress: an ERP study on Polish. Front. Psychol. 3:439. 10.3389/fpsyg.2012.00439 23125839 PMC3485581

[B16] Dupoux E. Sebastián-Gallés N. Navarrete E. Peperkamp S. (2008). Persistent stress “deafness”: the case of French learners of Spanish. Cognition 106, 682–706. 10.1016/j.cognition.2007.04.001 17592731

[B17] Ellis R. J. Jones M. R. (2010). Rhythmic context modulates foreperiod effects. Atten. Percept. Psychophys. 72, 2274–2288. 10.3758/BF03196701 21097869

[B18] Falk S. Dalla Bella S. (2016). It is better when expected: aligning verbal and motor rhythms enhances verbal processing. Lang. Cogn. Neurosci. 31, 699–708. 10.1080/23273798.2016.1144892

[B19] Falk S. Lanzilotti C. Schön D. (in press). Tuning neural phase entrainment to speech. J. Cogn. Neurosci. 10.1162/jocn_a_01136 28430043

[B20] Féry C. Ishihara S. (2008). How focus and givenness shape prosody, in Information Structure from Different Perspectives, eds Zimmerman M. Féry C.(Oxford: Oxford University Press), 36–63.

[B21] Féry C. Kügler F. (2008). Pitch accent scaling on given, new and focused constituents in German. J. Phon. 36, 680–703. 10.1016/j.wocn.2008.05.001

[B22] Fraisse P. Voillaume C. (1971). Les répères du sujet dans la synchronisation et dans la pseudo-synchronisation. L'Année Psychol. 71, 359–369. 10.3406/psy.1971.27747 5151289

[B23] Fujioka T. Zendel B. R. Ross B. (2010). Endogenous neuromagnetic activity for mental hierarchy of timing. J. Neurosci. 30, 3458–3466. 10.1523/JNEUROSCI.3086-09.2010 20203205 PMC6634108

[B24] Hayes B. (1995). Metrical Stress Theory: Principles and Case Studies. Chicago, IL: University of Chicago Press.

[B25] Holle H. Obermeier C. Schmidt-Kassow M. Friederici A. D. Ward J. Gunter T. C. (2012). Gesture facilitates the syntactic analysis of speech. Front. Psychol. 3:74. 10.3389/fpsyg.2012.00074 22457657 PMC3307377

[B26] Hommel B. Müsseler J. Aschersleben G. Prinz W. (2001). The Theory of Event Coding (TEC): a framework for perception and action planning. Behav. Brain Sci. 24, 849–878. 10.1017/S0140525X01000103 12239891

[B27] Ito T. Gracco V. L. Ostry D. J. (2014). Temporal factors affecting somatosensory-auditory interactions in speech processing. Front. Psychol. 5:1198. 10.3389/fpsyg.2014.01198 25452733 PMC4233986

[B28] Janata P. Tomic S. T. Haberman J. M. (2012). Sensorimotor coupling in music and the psychology of the groove. J. Exp. Psychol. 141, 54–75. 10.1037/a0024208 21767048

[B29] Jessen M. (1999). German, in Word Prosodic Systems in the Languages of Europe, ed van der Hulst H.(Berlin; New York, NY: Mouton de Gruyter), 515–544.

[B30] Jones M. Moynihan H. MacKenzie N. Puente J. (2002). Temporal aspects of stimulus-driven attending in dynamic arrays. Psychol. Sci. 13, 313–319. 10.1111/1467-9280.00458 12137133

[B31] Jun S.-A. Fougeron C. (2000). A phonological model of French intonation, in Intonation: Analysis, Modelling and Technology, ed Botinis A.(Boston, MA: Kluwer), 209–242.

[B32] Jun S.-A. Fougeron C. (2002). Realizations of accentual phrase in French intonation. Probus 14, 147–172. 10.1515/prbs.2002.002

[B33] Kelso J. A. Vatikiotis-Bateson E. Saltzman E. L. Kay B. (1985). A qualitative dynamic analysis of reiterant speech production: phase portraits, kinematics, and dynamic modeling. J. Acoust. Soc. Am. 77, 266–280. 10.1121/1.392268 3973219

[B34] Kotz S. A. Schwartze M. (2010). Cortical speech processing unplugged: a timely subcortico-cortical framework. Trends Cogn. Sci. 14, 392–399. 10.1016/j.tics.2010.06.005 20655802

[B35] Kotz S. A. Schwartze M. (2016). Motor timing and sequencing in speech production. A general-purpose framework, in Neurobiology of Language, eds Hickock G. Small S.(Amsterdam: Elsevier), 717–724.

[B36] Lakatos P. Karmos G. Mehta A. D. Ulbert I. Schroeder C. E. (2008). Entrainment of neuronal oscillations as a mechanism of attentional selection. Science 320, 110–113. 10.1126/science.1154735 18388295

[B37] Large E. W. Jones M. R. (1999). The dynamics of attending: how people track time-varying events. Psychol. Rev. 106, 119–159. 10.1037/0033-295X.106.1.119

[B38] Large E. W. Palmer C. (2002). Perceiving temporal regularity in music. Cogn. Sci. 26, 1–37. 10.1207/s15516709cog2601_1

[B39] Lehiste I. (1977). Isochrony reconsidered. J. Phon. 5, 253–263.

[B40] Liberman A. Prince A. (1977). On stress and linguistic rhythm. Linguist. Inq. 8, 249–336.

[B41] Lidji P. Palmer C. Peretz I. Morningstar M. (2011). Listeners feel the beat: entrainment to English and French speech rhythms. Psychon. Bull. Rev. 18, 1035–1041. 10.3758/s13423-011-0163-0 21912999 PMC3219863

[B42] London J. (2004). Hearing in Time. Oxford: Oxford University Press.

[B43] MacMillan N. A. Creelman C. D. (2005). Detection Theory. A User's Guide. Mahwah, NJ: Laurence Erlbaum.

[B44] Maes P.-J. Leman M. Palmer C. Wanderley M. M. (2014). Action-based effects on music perception. Front. Psychol. 4:1008. 10.3389/fpsyg.2013.01008 24454299 PMC3879531

[B45] Manning F. C. Schutz M. (2013). “Moving to the beat” improves timing perception. Psychon. Bull. Rev. 20, 1133–1139. 10.3758/s13423-013-0439-7 23670284

[B46] Manning F. C. Schutz M. (2016). Trained to keep a beat: movement-related enhancements to timing perception in percussionists and non-percussionists. Psychol. Res. 80, 532–542. 10.1007/s00426-015-0678-5 26067889

[B47] McNeill D. (1992). Hand and Mind: What Gestures Reveal About Thought. Chicago, IL: University of Chicago Press.

[B48] Mengel A. (2000). Deutscher Wortakzent. Norderstedt: Books on Demand.

[B49] Michelas A. D'Imperio M. (2015). Prosodic boundary strength guides syntactic parsing of French utterances. Lab. Phonol. 6, 119–146. 10.1515/lp-2015-0003

[B50] Michelas A. Frauenfelder U. H. Schön D. Dufour S. (2016). How deaf are French speakers to stress? J. Acoust. Soc. Am. 139, 1333–1342. 10.1121/1.4944574 27036270

[B51] Millotte S. Rene A. Wales R. Christophe A. (2008). Phonological phrase boundaries constrain the online syntactic analysis of spoken sentences. J. Exp. Psychol. Learn. Mem. Cogn. 34, 874–885. 10.1037/0278-7393.34.4.874 18605875

[B52] Morillon B. Hackett T. A. Kajikawa Y. Schroeder C. E. (2015). Predictive motor control of sensory dynamics in auditory active sensing. Curr. Opin. Neurobiol. 31, 230–238. 10.1016/j.conb.2014.12.005 25594376 PMC4898262

[B53] Morillon B. Schroeder C. E. Wyart V. (2014). Motor contributions to the temporal precision of auditory attention. Nat. Commun. 5:5255. 10.1038/ncomms6255 25314898 PMC4199392

[B54] Morton J. Martin S. M. Frankish C. (1976). Perceptual centers (P-centers). Psychol. Rev. 83, 405–408. 10.1037/0033-295X.83.5.405

[B55] Ong W. J. (2002). Orality and Literacy: The Technologizing of the Word. New York, NY: Routledge.

[B56] Opie I. Opie P. (1988). The Singing Game. Oxford: Oxford University Press.

[B57] Opie I. Opie P. (1997). The Oxford Dictionary of Rhymes. Oxford: Oxford University Press.

[B58] Otterbein S. Abel C. Heinemann L. V. Kaiser J. Schmidt-Kassow M. (2012). P3b reflects periodicity in linguistic sequences. PLoS ONE 7:e51419. 10.1371/journal.pone.0051419 23251527 PMC3519624

[B59] Parrell B. Goldstein L. Lee S. Byrd D. (2014). Spatiotemporal coupling between speech and manual motor actions. J. Phon. 42, 1–11. 10.1016/j.wocn.2013.11.002 24465063 PMC3900246

[B60] Peelle J. E. Gross J. Davis M. H. (2013). Phase-locked responses to speech in human auditory cortex are enhanced during comprehension. Cereb. Cortex 23, 1378–1387. 10.1093/cercor/bhs118 22610394 PMC3643716

[B61] Peperkamp S. Dupoux E. (2002). A typological study of stress 'deafness'. Lab. Phonol. 7, 203–240. 10.1515/9783110197105.203

[B62] Phillips-Silver J. Trainor L. J. (2005). Feeling the beat: movement influences infant rhythm perception. Science 308:1430. 10.1126/science.1110922 15933193

[B63] Pitt M. A. Samuel A. G. (1990). The use of rhythm in attending to speech. J. Exp. Psychol. Hum. Percept. Perform. 16, 564–573. 10.1037/0096-1523.16.3.564 2144571

[B64] Post B. M. B. (2000). Tonal and Phrasal Structures in French Intonation. The Hague: Thesus.

[B65] Prinz W. (1990). A common coding approach to perception and action, in Relationships Between Perception and Action: Current Approaches, eds Neumann O. Prinz W.(Berlin; Heidelberg: Springer), 167–201. 10.1007/978-3-642-75348-0_7

[B66] Quené H. Port R. F. (2005). Effects of timing regularity and metrical expectancy on spoken-word perception. Phonetica 62, 1–13. 10.1159/000087222 16116301

[B67] Repp B. H. (2003). Phase attraction in sensorimotor synchronization with auditory sequences: effects of single and periodic distractors on synchronization accuracy. J. Exp. Psychol. Hum. Percept. Perform. 29, 290–309. 10.1037/0096-1523.29.2.290 12760616

[B68] Repp B. H. (2005). Sensorimotor synchronization: a review of the tapping literature. Psychon. Bull. Rev. 12, 969–992. 10.3758/BF03206433 16615317

[B69] Repp B. H. (2008). Multiple temporal references in sensorimotor synchronization with metrical auditory sequences. Psychol. Res. 72, 79–98. 10.1007/s00426-006-0067-1 16786353

[B70] Repp B. H. Penel A. (2004). Rhythmic movement is attracted more strongly to auditory than to visual rhythms. Psychol. Res. 68, 252–270. 10.1007/s00426-003-0143-8 12955504

[B71] Repp B. H. Su Y.-H. (2013). Sensorimotor synchronization: a review of recent research (2006–2012). Psychon. Bull. Rev. 20, 403–452. 10.3758/s13423-012-0371-2 23397235

[B72] Rochet-Capellan A. Laboissière R. Galván A. Schwartz J. L. (2008). The speech focus position effect on jaw-finger coordination in a pointing task. J. Speech Lang. Hear. Res. 51, 1507–1521. 10.1044/1092-4388(2008/07-0173) 18695015

[B73] Rolland G. Lœvenbruck H. (2002). Characteristics of the accentual phrase in French: an acoustic, articulatory and perceptual study, in Proceedings of the Speech Prosody 2002, eds Bel B. Marlien I.(Aix-en-Provence), 611–614.

[B74] Roncaglia-Denissen M. P. Schmidt-Kassow M. Kotz S. A. (2013). Speech rhythm facilitates syntactic ambiguity resolution: ERP evidence. PLoS ONE 8:e56000. 10.1371/journal.pone.0056000 23409109 PMC3568096

[B75] Rossi M. (1980). Le français, langue sans accent? in L' accent en Français Contemporain, eds Fónagy I. Léon P. P.(Montréal, QC: Didier), 13–51.

[B76] Schmidt-Kassow M. Heinemann L. V. Abel C. Kaiser J. (2013). Auditory-motor synchronization facilitates attention allocation. Neuroimage 15, 101–106. 10.1016/j.neuroimage.2013.05.111 23732882

[B77] Schmidt-Kassow M. Rothermich K. Schwartze M. Kotz S. A. (2011). Did you get the beat? Late proficient French-German learners extract strong-weak patterns in tonal but not in linguistic sequences. Neuroimage 54, 568–576. 10.1016/j.neuroimage.2010.07.062 20692349

[B78] Schön D. Tillmann B. (2015). Short- and long-term rhythmic interventions: perspectives for language rehabilitation. Ann. N.Y. Acad. Sci. 1337, 32–39. 10.1111/nyas.12635 25773614

[B79] Schwartze M. Rothermich K. Schmidt-Kassow M. Kotz S. A. (2011). Temporal regularity effects on pre-attentive and attentive processing of deviance. Biol. Psychol. 87, 146–151. 10.1016/j.biopsycho.2011.02.021 21382437

[B80] Sowiński J. Dalla Bella S. (2013). Poor synchronization to the beat may result from deficient auditory-motor mapping. Neuropsychologia 51, 1952–1963. 10.1016/j.neuropsychologia.2013.06.027 23838002

[B81] Stern D. N. (1974). The goal and structure of mother-infant play. J. Am. Acad. Child. Psychiatry. 13, 402–421 10.1016/S0002-7138(09)61348-0 4427039

[B82] Sturt P. Sanford A. J. Stewart A. Dawydiak E. (2004). Linguistic focus and good-enough representations: an application of the change-detection paradigm. Psychon. Bull. Rev. 11, 882–888. 10.3758/BF03196716 15732698

[B83] Toro J. M. Sebastian-Gallès N. Mattys S. L. (2009). The role of perceptual salience during the segmentation of connected speech. Eur. J. Cogn. Psychol. 21, 786–800. 10.1080/09541440802405584

[B84] Vaissière J. (1991). Rhythm, accentuation and final lengthening in French, in Music, Language, Speech and Brain: Proceedings of an International Symposium at the Wenner-Gren Center, Stockholm, 5-8 September 1990, eds Sundberg J. Nord L. Carlson R.(London: MacMillan Education), 108–120.

[B85] van der Steen M. Keller P. E. (2013). The ADaptation and Anticipation Model (ADAM) of sensorimotor synchronization. Front. Hum. Neurosci. 7:253. 10.3389/fnhum.2013.00253 23772211 PMC3677131

[B86] Volman M. J. Geuze R. H. (2000). Temporal stability of rhythmic tapping “on” and “off the beat”: a developmental study. Psychol. Res. 63, 62–69. 10.1007/PL00008168 10743387

[B87] Vos P. G. Helsper E. L. (1992). Tracking simple rhythms: in-phase versus anti-phase performance, in Time, Action, and Cognition: Towards Bridging the Gap, eds Macar F. Pouthas V. Friedman W. J.(Dordrecht: Kluwer), 287–299.

[B88] Wan C. Y. Bazen L. Baars R. Libenson A. Zipse L. Zuk J.. (2011). Auditory-motor mapping training as an intervention to facilitate speech output in non-verbal children with autism: a proof of concept study. PLoS ONE 6:e25505. 10.1371/journal.pone.0025505 21980480 PMC3183050

[B89] Welby P. (2003). Effects of pitch accent position, type, and status on focus projection. Lang. Speech 46, 53–81. 10.1177/00238309030460010401 14529111

[B90] Welby P. (2006). French intonational structure: evidence from tonal alignment. J. Phon. 34, 343–371. 10.1016/j.wocn.2005.09.001

[B91] Welby P. (2007). The role of early fundamental frequency rises and elbows in French word segmentation. Speech Commun. 49, 28–48. 10.1016/j.specom.2006.10.005

[B92] Wing A. M. (2002). Voluntary timing and brain function: an information processing account. Brain Cogn. 48, 7–30. 10.1006/brcg.2001.1301 11812030

[B93] Wiese R. (2000). The Phonology of German. Oxford: Oxford University Press.

[B94] Zheng X. Pierrehumbert J. B. (2010). The effects of prosodic prominence and serial position on duration perception. J. Acoust. Soc. Am. 128, 851–859. 10.1121/1.3455796 20707454

